# Multiscale fusion network drives the repurposing of anticancer drugs

**DOI:** 10.1002/ctm2.1745

**Published:** 2024-06-25

**Authors:** Zhaoman Wan, Nan Jiang, Mingming Su, Xinlei Zhang, Yang Cao, Aiping Wu, Peng Zhang, Taijiao Jiang

**Affiliations:** ^1^ State Key Laboratory of Common Mechanism Research for Major Diseases, Suzhou Institute of Systems Medicine Chinese Academy of Medical Sciences & Peking Union Medical College Suzhou China; ^2^ 4+4 Medical Doctor Program Chinese Academy of Medical Sciences & Peking Union Medical College Beijing China; ^3^ Beijing Cloudna Technology Co., Ltd. Beijing China; ^4^ School of Biological Sciences Sichuan University Chengdu China; ^5^ Beijing Key Laboratory for Genetics of Birth Defects, Beijing Pediatric Research Institute, MOE Key Laboratory of Major Diseases in Children; Rare Disease Center, Beijing Children’s Hospital Capital Medical University, National Center for Children's Health Beijing China; ^6^ Guangzhou National Laboratory Guangzhou China

Dear Editor,

Drug repurposing is at the forefront of a transformative shift in computational methods driving new applications of approved or investigational drugs.^1^, ^2^ With the development of network pharmacology, repositioning algorithms for drug effects or drug targets are constantly expanding, but integrating multidimensional data to achieve precise repurposing is still a challenge.^3^, ^4^, ^5^, ^6^, ^7^, ^8^, ^9^ We focus on drug attribute characteristics and propose a scalable systematic paradigm. Using the Genomics of Drug Sensitivity in Cancer  (GDSC) database for anti‐tumor drugs, a integrated drug similarity network (iDSN) derived from different drug similarity networks (DSNs) based on chemical structure and drug target sequence data is constructed to infer potential drug pathways from drug properties and realise drug repurposing.

Initially, we processed drug profile data by vectorizing it (Figure 1A). Based on chemical and pharmacological properties, we constructed two separate DSNs: chem‐DSN and pharm‐DSN. These were then merged into an iDSN using a nonlinear fusion algorithm called Similarity Network Fusion (SNF) (Figure 1B). To validate the iDSN's potential in therapeutic similarity, we utilized a spectral clustering model with seven gold‐standard annotations from PubChem (Figure 1C). Downstream analysis was delineated across three dimensions for drug repurposing (Figure 1D): (1) identifying similar components within classes, amalgamating pharmacological mechanisms with pathway annotation; (2) establishing associations between drug network clusters and distinct biological pathways; (3) prioritizing higher‐ranked drug pairs for drug repositioning.

**FIGURE 1:**
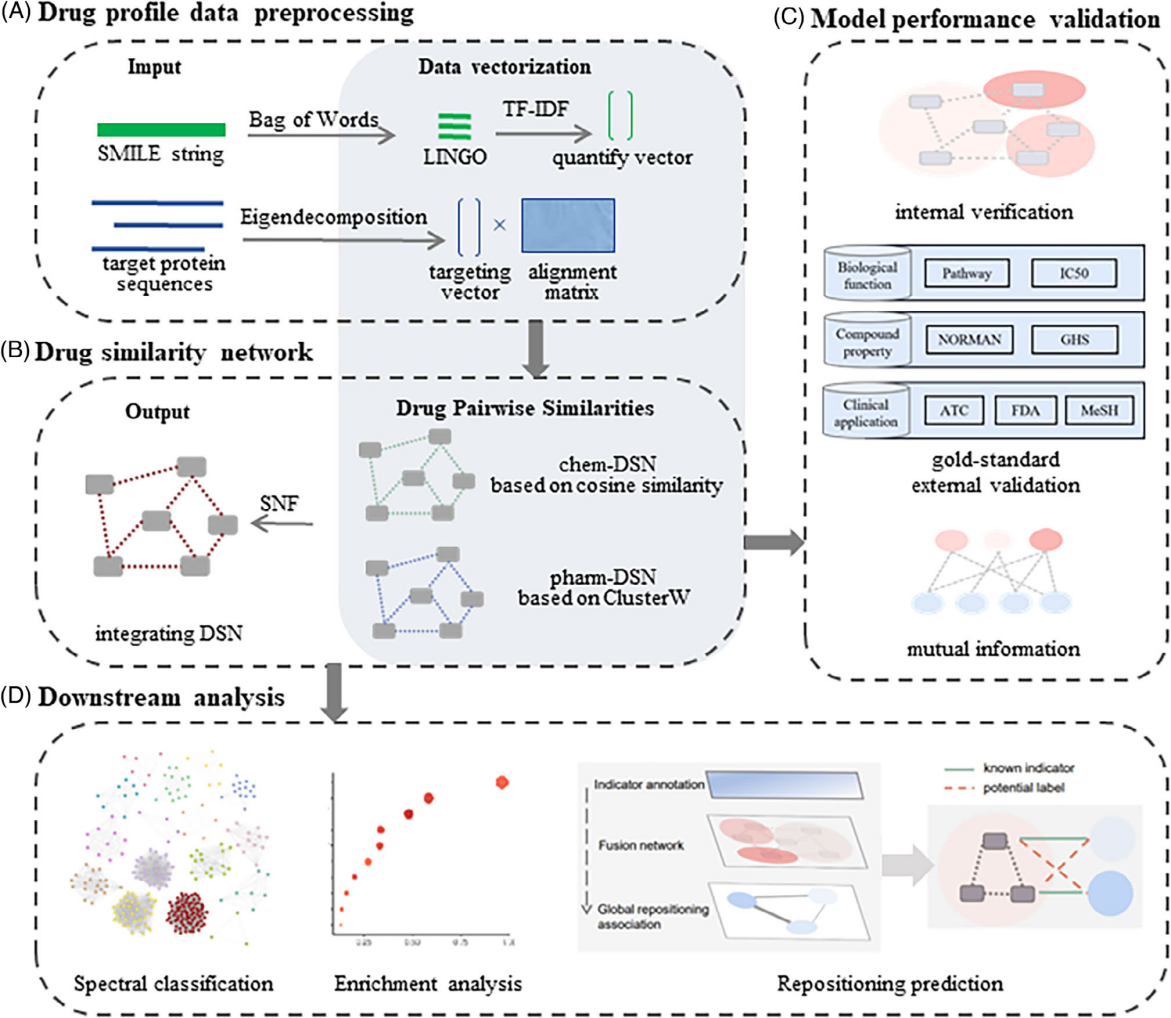
**The overall flowchart of the proposed framework**. (A) In the first part, we preprocess structural data and target protein sequences based on 276 anti‐tumor drugs containing target protein, PubChem CID and pathway annotations in GDSC to construct eigenvectors or eigen decomposition matrices that can be used for similarity network analysis. (B) In the second part, we constructed two structure‐based single‐property DSNs by vector space model and multiple sequence alignment denoted as chem‐DSN and pharm‐DSN, respectively, and fused them into multi‐scale network, named iDSN. (C) In the third part, we used the drug classification notes on DrugBank and PubChem as the gold standard to test the classification effect of DSN according to the adjusted Rand index. (D) In the fourth part, 276 drugs included in the iDSN were grouped into 16 clusters (Cluster 1–Cluster 16) and performed a systematic statistic analysis for annotating iDSN clusters from various perspectives to guide drug repositioning. Possible drug repositioning applications were obtained based on high‐similarity drug pairs or the occurrence of unexpected drugs within iDSN clusters.

Employing spectral clustering, iDSN exhibited a more distinct clustering structure compared to the chem‐DSN and a more evenly distributed structure than the pharm‐DSN (Figures 2A and S1). With the advantage of framework transparency, pharmacological properties contribute more than chemical properties through the quantitative assessment in clustering (Figure 2B). In 11 clusters, pharmacological features accounted for over 70% of edge similarity, and four clusters were entirely determined by pharm‐DSN. In comparison to single‐property DSNs, iDSN demonstrated superior performance across all three metrics (Figure 2C).

**FIGURE 2:**
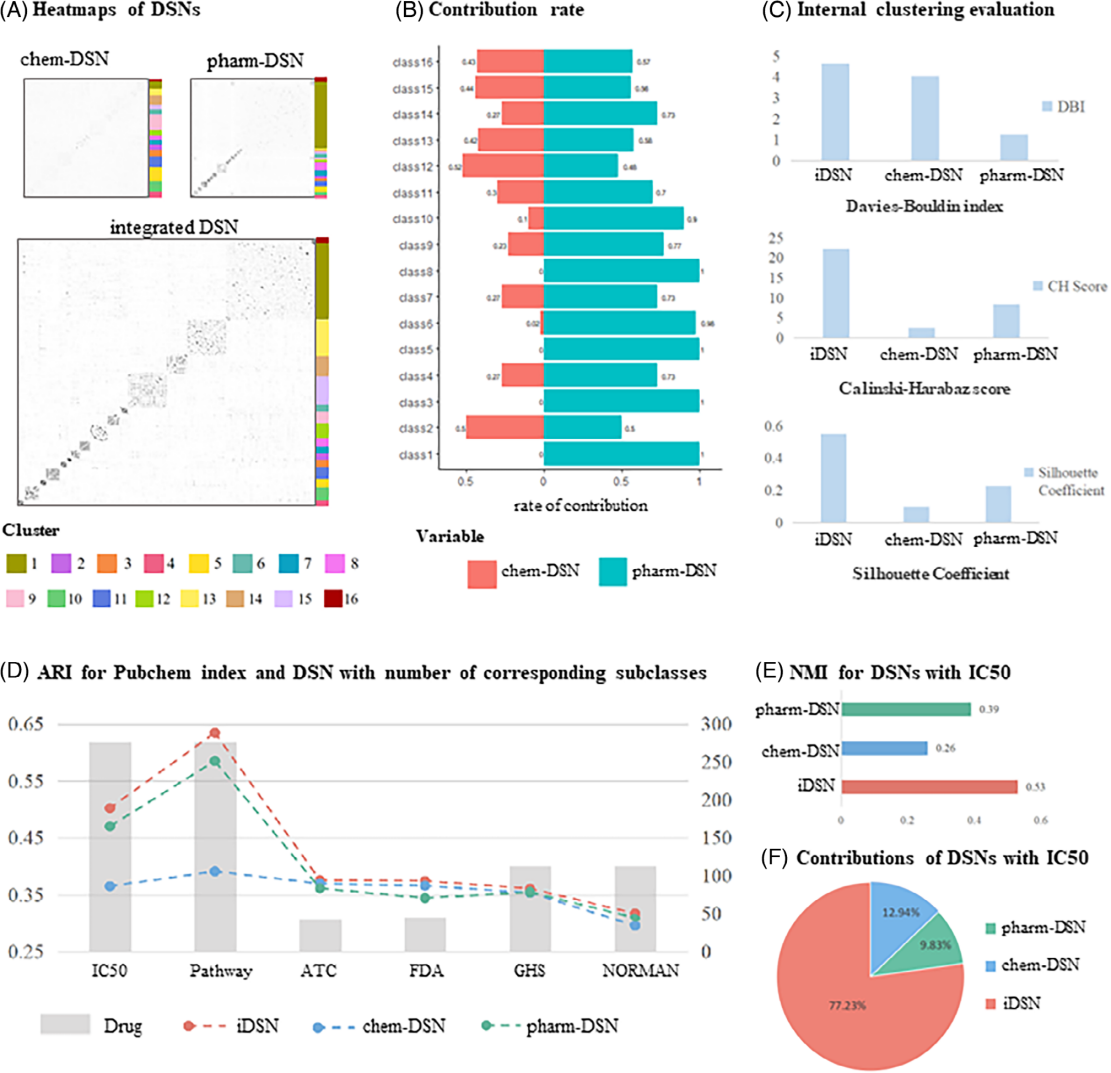
**Clustering validation and feature contribution of DSNs**. (A) Heatmaps of DSNs. chem‐DSN and pharm‐DSN were integrated into the iDSN. The sidebar to the left of the networks corresponds to the cluster label. (B) Structural features used alone were defined in the stem‐and‐leaf display with drug clusters to evaluate their respective contributions. (C) Categorical internal evaluation by Davies–Bouldin index, Calinski–Harabaz score and Silhouette coefficient for chem‐DSN, pharm‐DSN and iDSN. (D) Categorical external evaluation by adjusted RAND coefficient for chem‐DSN, pharm‐DSN and iDSN based on multiscale drug classification annotations. (E) Normalised mutual information bar chart of chem‐DSN, pharm‐DSN and iDSN with IC50‐based benchmark drug classification separately. (F) Data types contribution defined in the pie chart include chemical structure, drug targets and fusion value.

Evaluation using six diverse benchmark datasets of drug categories confirmed iDSN's stronger correlations with all benchmark annotations compared to single‐property DSNs (Table S1). Comparing clustering performance among single‐property DSNs, pharm‐DSN displayed better interaction with cell line (ARI = .470) and pathway (ARI = .585) annotations (Figure 2D). Importantly, iDSN based on the cross‐fusion network algorithm achieves higher performance on IC50 (ARI = .502, NMI = .53), indicating improved generalisation and accuracy through multi‐feature fusion (Figures 2E and S2). Data contribution analysis within the IC50‐based cluster of the three DSNs highlighted iDSN's predominant contribution (77.23%), while chem‐DSN and pharm‐DSN contributed less (9.83% and 12.94%, respectively), underscoring iDSN's dominance in the IC50‐based network (Figure 2F). To validate the superior performance, our method was compared with state‐of‐the‐art approaches, encompassing traditional machine learning, network propagation and matrix factorisation. The framework demonstrated a significantly higher value (SC = .58) compared to other methods. Similar results were observed with the NMI index using the IC50 dataset, where our framework outperformed in interactivity score (ARI = .512) (Table S2).

To facilitate drug precision repositioning, we uncovered the drug preferences within each cluster for various molecular functions using the KEGG pathway and Gene Ontology (GO) annotations^10^ (Figure 3). Notably, certain highly similar drug pairs within clusters exhibited consistent downstream pathway annotations, indicating the potential for drug repositioning by leveraging common targets or similar cellular signalling pathways to achieve therapeutic effects. The results revealed that several drug clusters exhibited significant enrichment annotations on GO analysis, such as Cluster 4, Cluster 2, and Cluster 5 included in the histone deacetylation, ADP ribosylation and phosphorylation respectively based on biological process. Furthermore, we observed that some individual drug clusters met different KEGG enrichment pathways under secondary classification, but specific on GO enrichment analysis in biological processes, cell components or molecular functions. For instance, in Cluster 9, we observed enrichment in KEGG pathways related to cancer and cell growth and death, with emphasis on apoptosis and molecular functions associated with dimerisation in biological processes, which suggests that Cluster 9 may exhibit a pharmacodynamic pattern, potentially influencing protein dimerisation and participating in pathways related to cancer or cell growth and death through apoptosis regulation.

**FIGURE 3:**
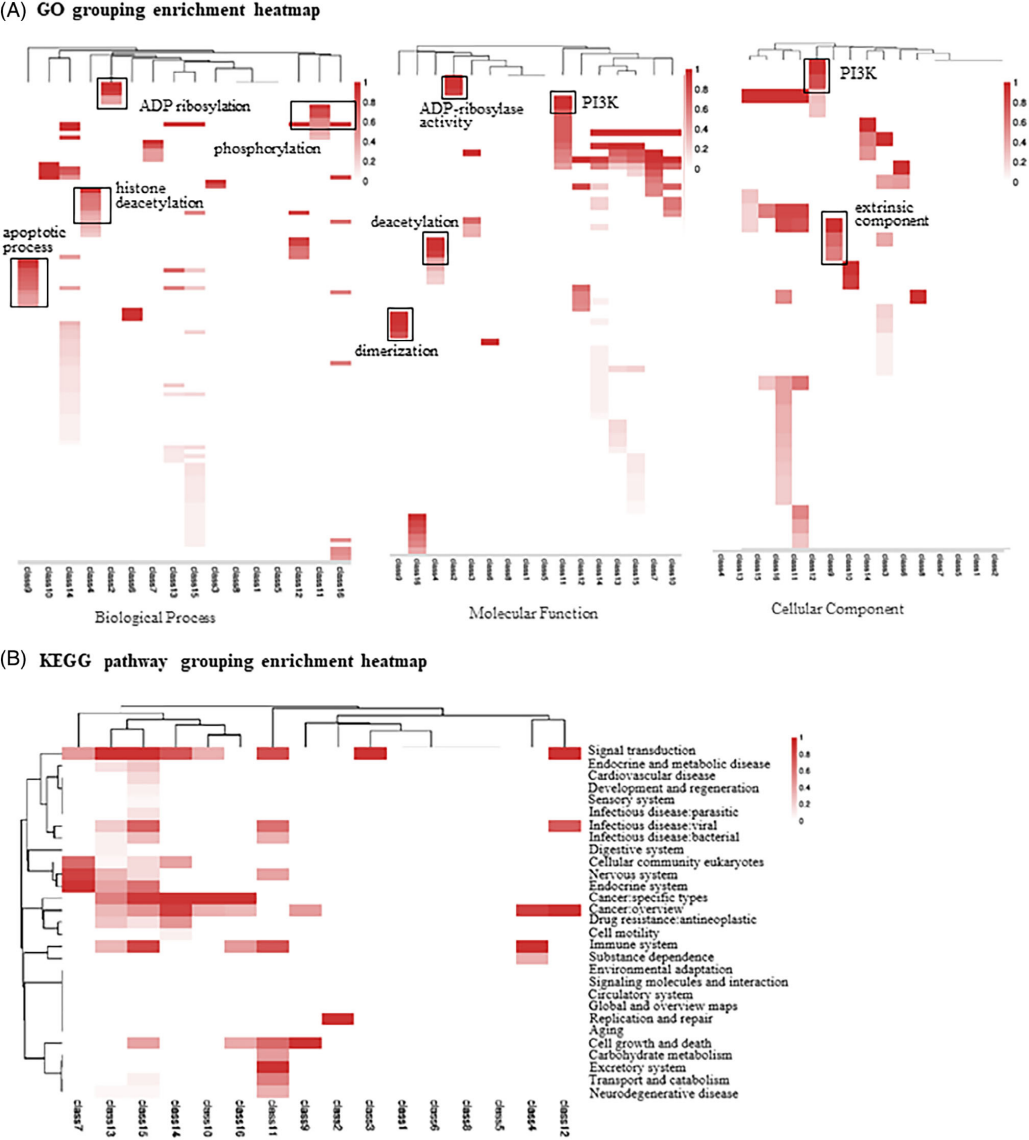
**Target‐based enrichment analyses**. (A) Enrichment landscape of GO biological processes, molecular function, and cellular components based on taxonomic clusters. (B) Enrichment landscape of pathway identifiers from the KEGG database. Deeper colour signifies greater significance in all panels, respectively. Common biological themes shared by multiple clusters are boxed with names provided in the plot.

Exploring drug pairs with high similarity in the iDSN reveals potential drug repositioning opportunities. For the top 100 similar drug pairs, 86% had consistent pathway annotations, confirming the reliability of our drug similarity calculations. However, some pairs with high similarity scores had different annotations, mainly linked to six pathways in four classification clusters (Figure 4A). For instance, a closely related set of drug pairs, including BMS‐536924, BMS‐754807, GSK1904529A, Linsitinib and NVP‐ADW742, exhibited connections to annotations in the IGF1R signalling and RTK signalling pathways, suggesting potential shared targets or interactions with similar cellular signalling pathways. In a major cluster, drugs associated with the kinases pathway (KIN001‐244) showed high similarity to drugs linked to the Metabolism pathway (BX‐912 and OSU‐03012) and the Mitosis pathway (MPS‐1‐IN‐1), unveiling potential crosstalk for therapeutic strategies (Table S3). Among them, the drug pair with the highest similarity is CMK‐LJI308 (ranked sixth), annotated in the kinase pathway and the PI3K/MTOR signalling pathway, respectively. Drug pairs with different annotation pathways in specific spectral clustering clusters indicate distinct subgroups with unique downstream pathway preferences. Some clusters may exhibit pathway‐specific therapeutic effects, while others show divergent pathway orientations (Figure 4B). To explore the global repositioning associations, we aligned pathway annotations with clustering results and observed a well‐balanced distribution of downstream pathways across drug clusters (Figure 4C). Within one cluster containing nine drugs, four were associated with the IGF1R signalling pathway, and the remaining drugs were linked to the RTK signalling pathway. In another cluster with 37 drugs, 14 were mapped to the RTK pathway, while the remaining drugs were connected to the kinase pathway. Furthermore, we independently analysed highly similar drug pairs within these clusters (Figure 4D).

**FIGURE 4:**
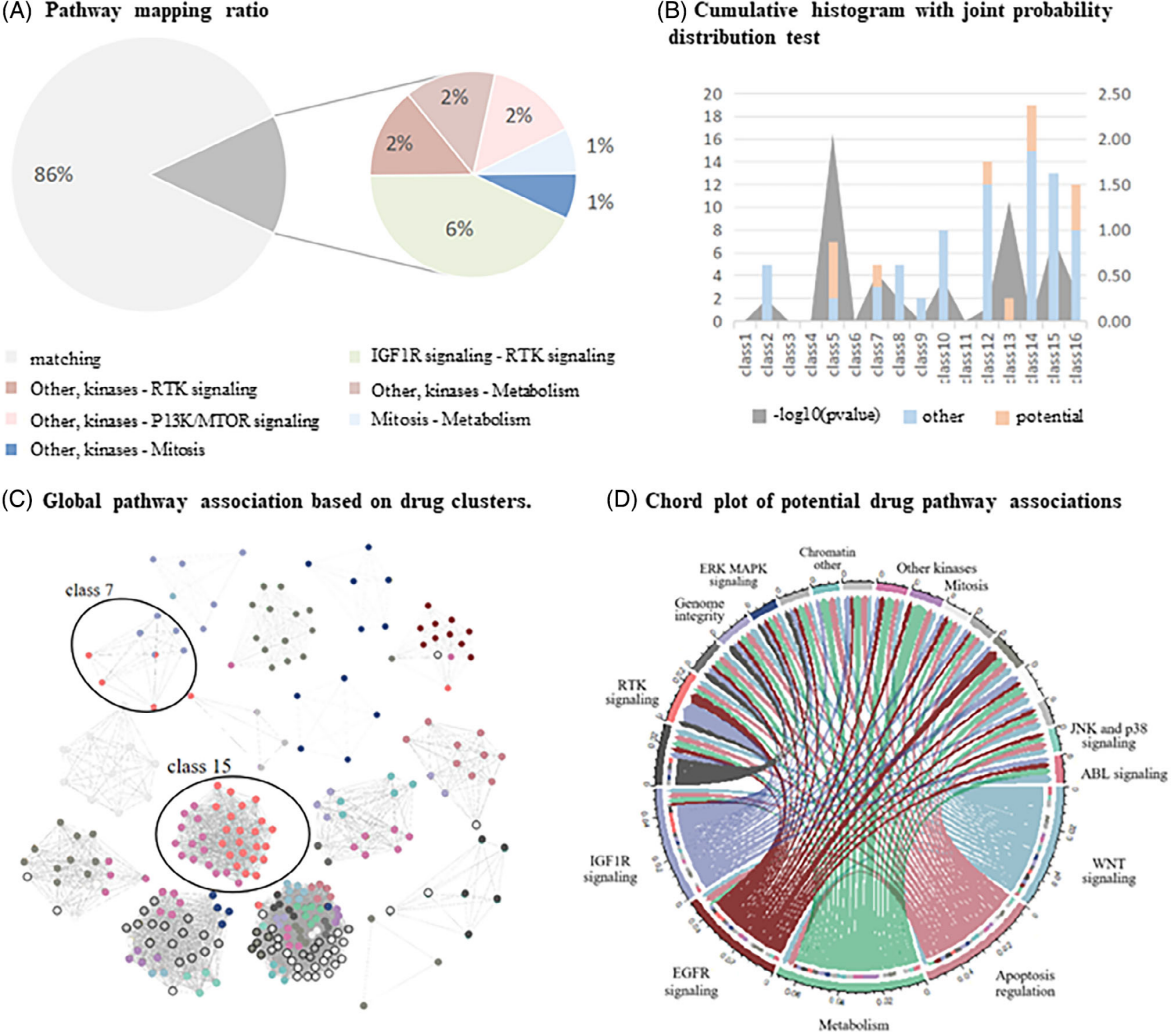
**Repositioning association with the different pathway**. (A) The consistent distribution of the top 100 similarity drug pairs on the pathway and the proportion of different pathway pairs. (B) The cumulative frequency histogram calculated the corresponding consistent pathway and nonconsistent pathway in the cluster under the distribution of the top 100 highly similar drug pairs in the drug cluster. The area plot represents whether the set of consistent and non‐consistent paths under each cluster evaluates the *p*‐value after taking the negative logarithm of the significance difference. The corresponding frequencies are represented by the left ordinate while the statistical test index is represented by the right. (C) The global anti‐tumor drugs in the iDSN are grouped into 16 clusters. Node colour signifies the pathway assignment of the drug. (D) Pathway association network for global drug mapping. The peripheral ring represents the GDSC drug annotation pathway, and the arc length is determined by the central angle corresponding to the proportion of the drug counts included in the pathway. The lines represent inconsistent pathways mapped under 276 drug global associations. The inner ring represents the potential pathway of the drug on the outer circumference of the same radius. The colour‐indicated pathways correspond to inconsistent pathway‐source drug pairs with a similarity greater than .7, and the grey‐indicated pathways correspond to highly similar drugs that are all mapped on the same pathway.

In conclusion, this scalable structure‐derived framework offers fresh insights into deducing characteristic downstream pathways and repurposing drugs via common drug structural properties. With the accumulation of drug informatics data and the development of future drugs, we will continue to expand our data, to deepen our understanding of feature integration, and to further improve the algorithm's performance for new drug development.

## AUTHOR CONTRIBUTIONS

Zhaoman Wan performed the analysis and prepared the manuscript with the help of Yang Cao, Mingming Su, Xinlei Zhang, L.Y. and H.X. Aiping Wu, Peng Zhang and Taijiao Jiang supervised the studies, designed the analysis and revised the manuscript. All authors reviewed and approved the manuscript.

## CONFLICT OF INTERESTS STATEMENT

M.S. and X.Z. are the co‐founders of Beijing Cloudna Technology Co., Ltd., and the other authors declare no competing interests.

## Supporting information

Supporting Information

Supporting Information

Supporting Information

Supporting Information

Supporting Information

## Data Availability

The materials of datasets used for the current study were accessible in the specific public database (as we described in the Methods section). The analysis codes are available at https://github.com/zmwwan/DSNcode/.
