# Supplementary material for: Multiscale fusion network drives the repurposing of anticancer drugs
Source: Clin Transl Med. 2024 Jun 25;14(7):e1745. doi: 10.1002/ctm2.1745 (PMC11199060; doi:10.1002/ctm2.1745)
Supplement: Supplementary file 1 — Supporting Information [file CTM2-14-e1745-s002.pptx]

## Slide 1
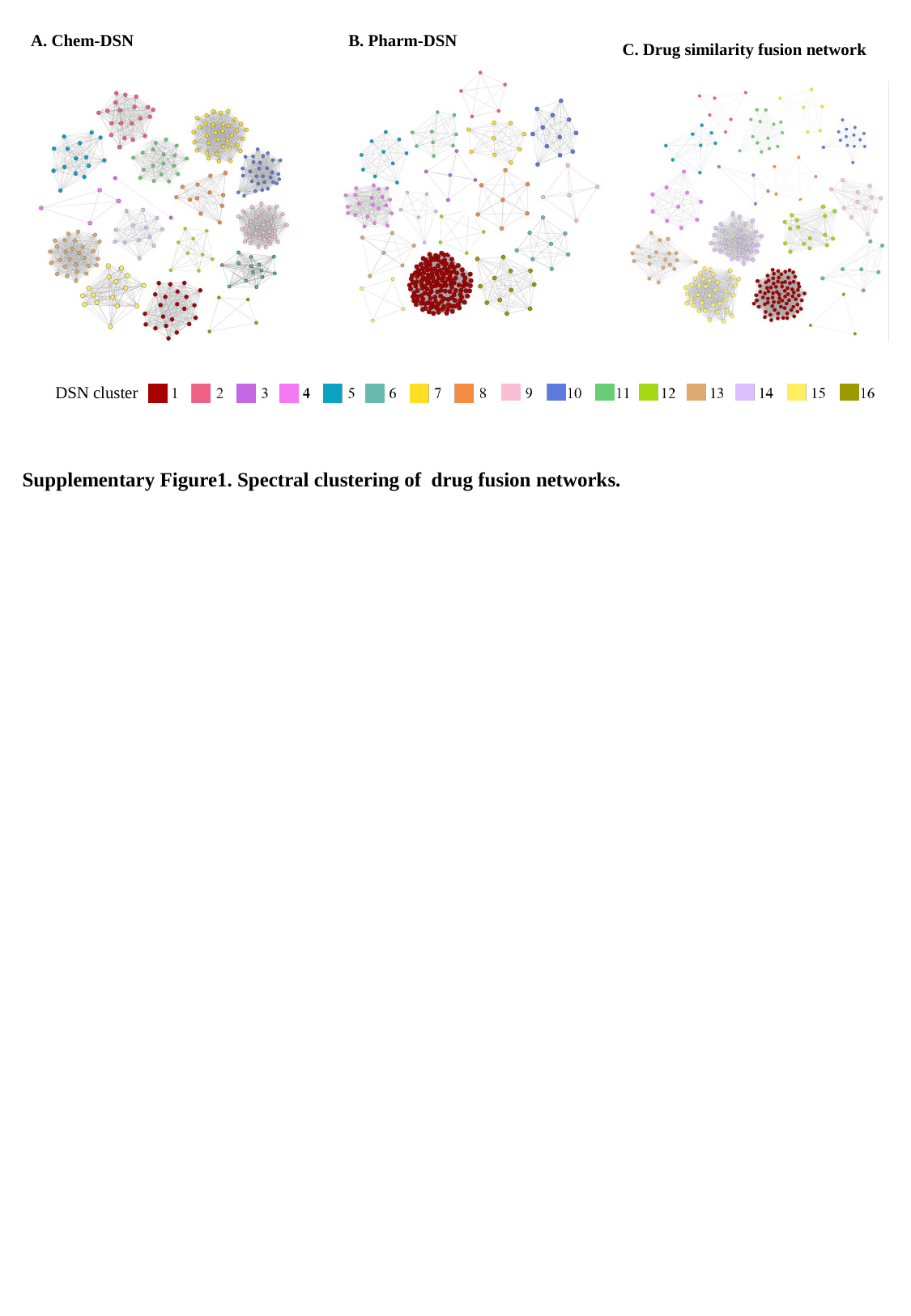

A. Chem-DSN
B. Pharm-DSN
C. Drug similarity fusion network
DSN cluster
Supplementary Figure1. Spectral clustering of drug fusion networks.

## Slide 2
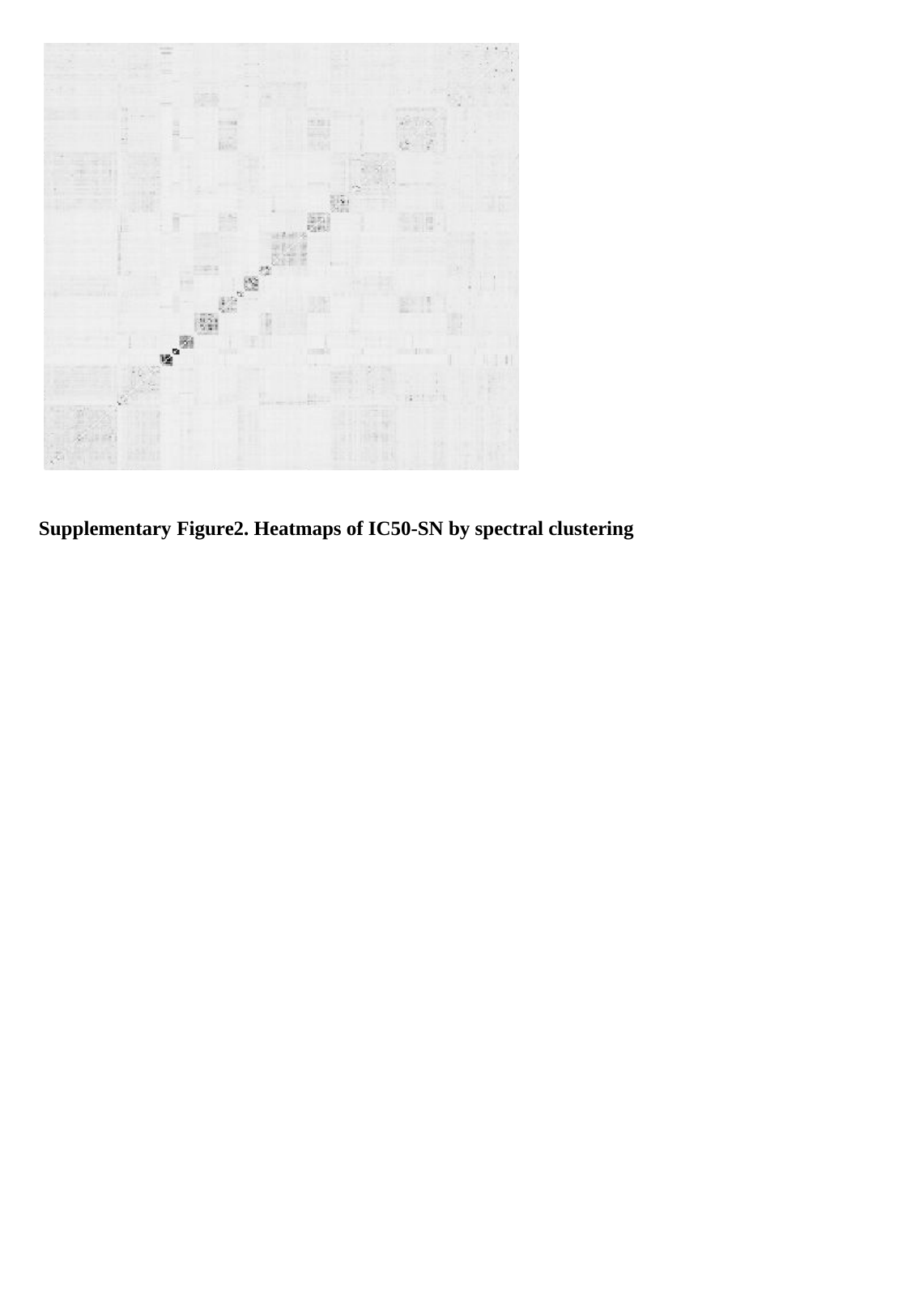

Supplementary Figure2. Heatmaps of IC50-SN by spectral clustering
