# Supplementary material for: Multiscale fusion network drives the repurposing of anticancer drugs
Source: Clin Transl Med. 2024 Jun 25;14(7):e1745. doi: 10.1002/ctm2.1745 (PMC11199060; doi:10.1002/ctm2.1745)
Supplement: Supplementary file 2 — Supporting Information [file CTM2-14-e1745-s001.docx]

**Materials and Methods**

**Datasets access and preprocessing**

**Data source**

We collected information on 542 anticancer drugs from the GDSC database, a bioinformatics resource with drug sensitivity in cancer cells and molecular markers of drug response (Table 1). Protein sequence data were extracted from the UniProt database, which provides high-quality, freely accessible protein sequence data [1]. We extracted canonical SMILES of drugs from PubChem Compound database, which contains more than 92 million unique structures of compounds [2].

**Data preprocessing**

We first vectorized the structural data of the drug and the corresponding targeted protein. The original drug structure is represented as a SMILES (Simplified Molecular Input Line Entry System) string, which uses ASCII characters to encode the structural and chemical information of the molecule [3,4]. In order to quantitatively measure the similarity between drugs, we used the TF-IDF algorithm to construct a Bag of Words model and converted n-character SMILES texts into q-character substrings with$(n - (q - 1))$ length LINGO representation [5]. Thus, for each dimensional phrase, term frequency (TF) and inverse document frequency (IDF) of LINGO L in SMILES string S calculated as follows:

$$\begin{matrix} w_{\mathrm{TF}}=\{\begin{matrix} 1+\log_{10} F(L_{i},S), if F(L_{i},S)>0 \\ 0 , otherwise \end{matrix} \\ w_{\mathrm{IDF}}=\log_{10} \frac{N}{\left| L\in D, L_{i}\in L \right|} \\ \end{matrix}$$

$F(L,S)$ reflects the frequency of times a LINGO substring occurs in a SMILES text. Substring corpus, and number of SMILES text in the corpus, respectively. Further, the TF-IDF weighting-scheme $W={(w_{1},...,w_{n - (q - 1)})}^{T}$ assigns higher weights to those terms that occur frequently in a SMILE text while rarely in all collections , where $w_{i}=w_{TF}\cdot w_{IDF}$.

At the same time, we summarized the corresponding target proteins of the drug set and constructed a drug target vector based on the sequence features of each drug profile represented by a logical vector $\mathbf{T}={(t_{1},...,t_{n})}^{T}$, where is the number of targeted protein. Each element in the drug profile can be written in the following index form:

$$t_{i}=\{\begin{matrix} 1, if ith protein is observed \\ 0 , \mathrm{otherwise} \end{matrix}$$

**Construction of individual drug similarity networks / Drug pairwise similarities**

We defined and computed two drug–drug similarity measures under structure properties separately. Molecular-based similarity for drug pair is calculated according to the cosine angle between their TF-IDF weighting:

$$\mathrm{Sim}_{\mathrm{molecular}}=\frac{\sum_{i=1}^{(n - (q - 1))} w_{L_{i},S_{1}}\cdot w_{L_{i},S_{2}}}{\left\| w_{L_{i},S_{1}} \right\|\cdot\left\| w_{L_{i},S_{2}} \right\|}$$

The target-based similarity of a drug pair is decomposed into the proportion of the same target protein and the sequence similarity rank between different proteins. The latter is obtained by decomposing the target protein similarity network $S$ evaluated by ClusterW algorithm based on the feature vector $T$, where each weight element represents the similarity score between protein sequence pairs calculated based on Smith-Waterman.

$$\mathrm{Sim}_{\mathrm{target}}=\frac{\left\| T_{1}T_{2}^{'} \right\|+T_{1}ST_{2}^{'}}{\left\| T_{1} \right\|\cdot\left\| T_{2} \right\|}$$

By exploring the intrinsic similarity connections between pairs of drugs, our methodology enables the creation of two autonomous drug similarity networks. These two independent networks provide distinct perspectives on drug similarity by elucidating relationships based on different properties. The molecular-based network (chem-DSN), rooted in drug attribute structure, unveils similarities arising from chemical and biological characteristics. On the other hand, the target-based network (pharm-DSN), anchored in drug target protein structure, offered insights into similarities driven by the underlying protein interactions. Together, these parallel networks offer a nuanced understanding of drug similarity, encompassing diverse facets of pharmacological and biological relevance.

**Structural neighborhood fusion (SNF)**

To construct the drug similarity network using the Similarity Network Fusion (SNF) algorithm, which enhances robustness to noise by emphasizing similarities rather than raw data values, and help preserve both fine-scale and large-scale structures within and between datasets. We employed the pairwise relationships between drugs in both the structural and targeted domains from collect and preprocess data to ensure the seamless integration of chem-DSN and pharm-DSN, revealing a comprehensive perspective on the multifaceted relationships within the realm of drug similarities [6,7].

First we normalized the outputs from the SNF algorithm to ensure consistency and comparability between the two networks. To different dimensional measurement characteristics, all similarity measures were normalized to be in the range [0, 1]. Subsequently, we fused the normalized DSN into a unified drug similarity network on affinity pattern by exponential similarity

$$e(i,j)=exp(-\frac{\rho^{2}(s_{i},s_{j})}{\mu\varepsilon_{s_{i},s_{j}}})$$

where is a hyperparameter that we empirically set 0.5, and is used to eliminate the scaling problem [20]. $e(i,j)$ utilized the principles of similarity network fusion to integrate the structural and targeted drug similarity networks, revealing shared information and potential correlation.

Finally, we encapsulated the synergistic information from both structural and targeted domains to construct the standardized graph fusion affinity by unifying them into a cohesive network using the principles of similarity network fusion.

$$\mathrm{Sim}_{\mathrm{fusion}}=\left\{ \begin{matrix} \frac{e(s_{i},s_{j})}{2\sum_{s_{k}\neq s_{i}} e(s_{i},s_{k})}, i\neq j \\ 0.5, i=j \end{matrix} \right.$$

**Visualization and downstream analysis**

First, we visualized the constructed drug similarity network and grouped drugs with similar structural or functional characteristics into the same cluster by spectral clustering to comprehend the interconnections between drugs and identify groups of drugs with similar mechanisms of action or therapeutic effects. Then, by combining known biological information and databases, we annotated the function of drugs and their potential cell line and pathways to help understand the mechanism of drug action and predict new indications. We performed pathway-enrichment analyses including 206 biological pathways in the following Kyoto Encyclopedia of Genes and Genomes (KEGG) categories: metabolism, environmental information processing, cellular processes and organismal systems [8]. For each cluster, we performed Gene Ontology (GO) enrichment analyses based on the International Union of Basic and Clinical Pharmacology/British Pharmacological Society (IUPHAR/BPS). KEGG and GO enrichment are tested by 0.05 threshold of p-adjusted with the ClusterProfiler tool in R software.

For the structure-based drug repurposing analysis, we derived potential applications of drugs to other mechanisms for every drug from two perspectives with the following procedures. Based on the fusion DSN, we extract drug pair ranked in the top 100 in the fusion similarity score as potential liquidity drugs. Second, we select high similarity drug pair with differ pathway labels. Each unmarked pathway in the drug pair was defined as the potential repurposing ability in the drug-expression therapeutic across drug pair. Based on drug clustering, we hypothesize that intra-cluster drugs have a credible pathway interaction. We evaluated the probability of observing the repopursing of inner-cluster drugs in a pathway map, and focus on drug clusters with approximately uniform distribution pathways. Finally, we predicted the drug repopuring for each cluster on the inferred DSN by connecting the drug-similarity pathway patterns.

**Automatic learning packaging for drug repurposing**

We encapsulate the above process as an automated process that aims to streamline the identification of potential drug repositioning candidates by integrating diverse drug information and leveraging advanced network analysis techniques, facilitating targeted and precise drug repositioning strategies. The process involves the following key steps:

**Input:** Utilize two types of drug information: chemical structures (SMILES-based compounds from PubChem) and molecular targets (protein target sequence data from UniProt).

**Step1:** Drug similarity: Create two Drug Similarity Networks (DSNs): chem-DSN based on molecular property similarity using TF-IDF weighting and pharm-DSN based on pharmacological property similarity using Clusterw global comparison.

**Stpe2:** Apply the Spectral Network Fusion (SNF) algorithm to nonlinearly combine chem-DSN and pharm-DSN into an integrated Drug Similarity Network (iDSN).

**Stpe3:** Employ spectral clustering on the iDSN to identify clusters of drugs with similar therapeutic properties, and perform target-based enrichment analysis to label and annotate each cluster, confirming the therapeutic similarity of drugs within clusters.

**Output:** Identify unexpected drug pairs with high similarity but inconsistent pathway labels within each cluster.

**Evaluation Metrics**

The test statistic, Silhouette Coefficient Index, Calinski-Harabasz Index, and Davies-Bouldin Index are used for internal verification to reflect the rationality of drug clusters based on relative similarity between drugs.

Silhouette coefficient measures the ratio of the average distance of the intra-cluster drug pair and the nearest out-of-cluster drug pair distance, with a smaller index indicating better clustering. The silhouette coefficient of the overall clustering effect is calculated as follows:

$$SC=\frac{1}{n}\sum_{i=1}^{n} \frac{b_{i}-a_{i}}{\max\left\{ a_{i},b_{i} \right\}}$$

where n is the number of drugs, is the mean value between drug and all other drugs within the same cluster, is the lowest distance of drug to all points in any other clusters.

More intuitively, the Calinski-Harabasz index measures the ratio of inter-cluster distance to intra-cluster distance, with a higher index indicating better clustering, and its formula is similar to the variance calculation as follows:

$$CH=\frac{n-K}{K-1}\cdot\frac{\sum_{k=1}^{K} {n_{k}(c_{k}-c)}^{2}}{\sum_{k=1}^{K} \sum_{d\in C_{k}} {(d-c_{k})}^{2}}$$

where represent the global cluster center, the number of drugs, and the cluster, respectively. represents all drugs in cluster, and represents the cluster center corresponding to cluster.

Davies-Bouldin Index calculates the average similarity between each cluster and its most similar cluster to measure the overall clustering with a higher index indicating better clustering. The Davies-Bouldin Index based on the similarity definition calculates as follows:

$$DB=\frac{1}{K}\sum_{i,j=1}^{K} \max_{i\neq j} \frac{s_{i}+s_{j}}{d_{\mathrm{ij}}}$$

where is the mean average distance of intra-cluster diameter, represents the distance between the centers of cluster and cluster.

**Benchmark test**

We extracted 4 gold standard annotations downloaded from the PubChem database to measure the overlap ratio with drug classification. Mutual information external validation, relying on Adjusted Rand Index (ARI) and Normalized Mutual Information (NMI), provides a comprehensive assessment of clustering performance by comparing the agreement between the obtained clustering results and the ground reference clustering.

Assuming the classification obeys random hypergeometry, Adjusted Rand index (ARI) measures the overlap between instance classification and cluster classification by contingency table as follow:

$$ARI=\frac{\sum_{\mathrm{ij}} (\begin{matrix} n_{\mathrm{ij}} \\ 2 \end{matrix})-{[\sum_{i} (\begin{matrix} a_{i} \\ 2 \end{matrix})\sum_{j} (\begin{matrix} b_{j} \\ 2 \end{matrix})]}/{(\begin{matrix} n \\ 2 \end{matrix})}}{\frac{1}{2}[\sum_{i} (\begin{matrix} a_{i} \\ 2 \end{matrix})+\sum_{j} (\begin{matrix} b_{j} \\ 2 \end{matrix})]-{[\sum_{i} (\begin{matrix} a_{i} \\ 2 \end{matrix})\sum_{j} (\begin{matrix} b_{j} \\ 2 \end{matrix})]}/{(\begin{matrix} n \\ 2 \end{matrix})}}$$

where and represent the number of drugs in annotation and classification, respectively. is the number of drug intersection between annotation and classification. ARI ranges from -1 to 1, where 1 indicates perfect agreement between the two clusterings, 0 indicates random clustering, and negative values indicate disagreement.

For the cell line dataset, we applied spectral clustering to divide the IC50 tensor into 16 matrices of the same size as the DSN. The normalized mutual information (NMI) measuring the mutual dependence between the IC50 label vector and the drug clustering label vector is calculated as follows:

$$NMI(\Omega,X)=\frac{I(\Omega;X)}{{(H(\Omega)+H(X))}/2}$$

Standing for mutual information and entropy, respectively, and is defined as

$$I(\Omega;X)=\sum_{k} \sum_{j} P(w_{k}\cap x_{j})\log\frac{P(w_{k}\cap x_{j})}{P(w_{k})P(x_{j})}$$

$$H(\Omega)=-\sum_{k} \frac{\left| w_{k} \right|}{N}\log\frac{\left| w_{k} \right|}{N}$$

where $P(w_{k})$, respectively represent the marginal probability distribution function of IC50 label vector and the drug clustering label vector $X$, and is the joint probability function of and $X$. Like ARI, NMI ranges from 0 to 1, where 1 indicates perfect agreement between the clusterings and 0 indicates no mutual information.

We summarized four different types of popular and competitive baseline to compare with our framework: 1 traditional machine learning; 2) based on network propagation; 3) based on matrix factorization prediction methods; 4) ensemble-based learning..For reproduced models, we focus on the SC index to internally verify DSNs discrimination by spectral clustering, and calculate NMI index to externally verify the information interaction between different DSNs and IC50 indicators.

**References**

1. UniProt Consortium. UniProt: a hub for protein information. Nucleic Acids Res. 2015 Jan;43(Database issue):D204-212.
2. Blustajn J, Thomas P, Combes C, Gaston A. Quel est votre diagnostic? [What is your diagnosis?]. J Neuroradiol. 1998 Oct;25(3):184-187.
3. Kumar A, Zhang KYJ. Advances in the Development of Shape Similarity Methods and Their Application in Drug Discovery. Front Chem. 2018; Jul 25; 6:315.
4. Hattori M, Tanaka N, Kanehisa M, Goto S. SIMCOMP/SUBCOMP: chemical structure search servers for network analyses. Nucleic Acids Res. 2010 Jul; 38: W652-656.
5. Öztürk H, Ozkirimli E, Özgür A. A comparative study of SMILES-based compound similarity functions for drug-target interaction prediction. BMC Bioinformatics. 2016 Mar 18;17:128.
6. Wang S, Li J, Wang D, Xu D, Jin J, Wang Y. Predicting Drug-Disease Associations Through Similarity Network Fusion and Multi-View Feature Projection Representation. IEEE J Biomed Health Inform. 2023 Oct;27(10):5165-5176.
7. He S, Wen Y, Yang X, Liu Z, Song X, Huang X, Bo X. PIMD: An Integrative Approach for Drug Repositioning Using Multiple Characterization Fusion. Genomics Proteomics Bioinformatics. 2020 Oct;18(5):565-581.
8. Kanehisa M, Goto S. KEGG: kyoto encyclopedia of genes and genomes. Nucleic Acids Res. 2000 Jan 1;28(1):27-30.
